# Testing Pairwise Association between Spatially Autocorrelated Variables: A New Approach Using Surrogate Lattice Data

**DOI:** 10.1371/journal.pone.0048766

**Published:** 2012-11-07

**Authors:** Vincent Deblauwe, Pol Kennel, Pierre Couteron

**Affiliations:** 1 Institut de Recherche pour le Développement, UMR AMAP, Montpellier, France; 2 Laboratoire d'Informatique, de Robotique et de Microélectronique de Montpellier, UMR 5506 – CC 477, Montpellier, France; 3 Centre de coopération internationale en recherche agronomique pour le développement, UMR AMAP, Montpellier, France; The Ohio State University, United States of America

## Abstract

**Background:**

Independence between observations is a standard prerequisite of traditional statistical tests of association. This condition is, however, violated when autocorrelation is present within the data. In the case of variables that are regularly sampled in space (i.e. lattice data or images), such as those provided by remote-sensing or geographical databases, this problem is particularly acute. Because analytic derivation of the null probability distribution of the test statistic (e.g. Pearson's r) is not always possible when autocorrelation is present, we propose instead the use of a Monte Carlo simulation with surrogate data.

**Methodology/Principal Findings:**

The null hypothesis that two observed mapped variables are the result of independent pattern generating processes is tested here by generating sets of random image data while preserving the autocorrelation function of the original images. Surrogates are generated by matching the dual-tree complex wavelet spectra (and hence the autocorrelation functions) of white noise images with the spectra of the original images. The generated images can then be used to build the probability distribution function of any statistic of association under the null hypothesis. We demonstrate the validity of a statistical test of association based on these surrogates with both actual and synthetic data and compare it with a corrected parametric test and three existing methods that generate surrogates (randomization, random rotations and shifts, and iterative amplitude adjusted Fourier transform). Type I error control was excellent, even with strong and long-range autocorrelation, which is not the case for alternative methods.

**Conclusions/Significance:**

The wavelet-based surrogates are particularly appropriate in cases where autocorrelation appears at all scales or is direction-dependent (anisotropy). We explore the potential of the method for association tests involving a lattice of binary data and discuss its potential for validation of species distribution models. An implementation of the method in Java for the generation of wavelet-based surrogates is available online as supporting material.

## Introduction

A major goal in natural sciences is to uncover and quantify the processes responsible for the spatial and temporal dynamics of natural systems. Depending on the spatial and temporal scales at which the processes act, manipulative experiments – that could provide repeated realizations of the process under controlled conditions – may be difficult if not impossible. As a consequence, a common practice consists in applying appropriate analytical tools to infer properties of the processes under study only from their available realizations, i.e. the observed natural patterns. Such an approach is all the more timely now that an impressive number of physical and biological variables are measured and mapped at global scales and at high resolutions (e.g. [1]). The increasing availability of these large gridded datasets is due not only to the advances in remote-sensing, geodesy, and information technologies, but also to numerous initiatives facilitating their accessibility. Among others, these are, for optical and biophysical variables, the Global Land Cover Facility (www.landcover.org) and the Google Earth Engine© platform, and, for organisms' occurrences, the Global Biodiversity Information Facility (www.gbif.org). In ecology, for instance, this broadens the use of spatial patterns or spatial residuals to uncover unmeasured or unmeasurable processes [2]. This approach has come to be used increasingly in ecology for the delimitation of environmental niches of species and the prediction of their geographic distributions [3]; in epidemiology for the control and forecasting of disease risks (see Hay *et al.* [4] for a dedicated volume) as well as in the social and environmental sciences for the description and understanding of processes that generate observable patterns [2], [5].

However, the very general and long standing question of determining whether two spatial patterns appear associated by chance or not still presents some unsettled problems. For instance, association tests may compare a species' abundance pattern or disease prevalence with the pattern of abundance of another species or of background environmental conditions, or with specific outputs of mechanistic models [6]–[8]. These examples are typical cases where the constraint on data collection does not commonly allow for recording repeated realizations of the hypothetical underlying process creating the association. We therefore resort to the principles of statistics to determine, under particular assumptions, the level of confidence we can grant to the observed measure of association.

Independence between individual observations is a usual assumption of traditional statistical tests, which is not guaranteed for any dataset that is not the product of truly random sampling. Because of the continuous nature of geographic variations (Tobler's “first law of geography” [9]), some correlation, positive at the shortest distances and decreasing as distance increases, is often present between spatialized observations. Other types of structures, for instance displaying negative autocorrelation at short range (inhibition), though less frequently reported, also exist. Whatever the structure, ignoring this spatial autocorrelation (SA) in statistical inferences means treating correlated observations as independent replicates, and thus is a form of pseudoreplication against which criticism has long been directed (for instance see [10], in ecology).

SA may arise at a wide range of scales for a number of reasons. For instance, in the distribution of organisms, SA may result from environmental, physiographical and historical factors that limit the mobility of organisms. Behavioural factors and other intrinsic dispersal limitation factors may also cause the spatial aggregation of populations and species in the landscape. The environment itself, which constrains the survival and reproduction of organisms, is also subject to SA. In addition to these intrinsic factors, SA can artificially arise in distribution data if the sampling effort is not constant over the studied area. We will not review here the different methods to test for the presence and magnitude of SA in data, but rather signal to the reader the large body of literature concerned with this issue [11].

When dealing with the association between variables, it is well known that the consequence of ignoring SA, and therefore violating the assumption of independence between observations, means an inflation in type I error rate (probability of falsely rejecting the null hypothesis, usually denoted by α) that is an increasing function of the degree of SA [12], [13]. Several alternatives to standard statistical approaches have been proposed so as to provide unbiased coefficient estimation in regression analysis [11], [14]–[16] or for the study of scale-specific associations [17]–[19]. There is, however, no general framework for the analysis and statistical inference of association between autocorrelated variables.

Our aim here is to propose an unbiased Monte-Carlo test for lattice or image data based on the generation of sets of “surrogate” data that share the autocorrelation function of observed data. We introduce a new generation method based on an image synthesis technique using a particular class of wavelet transform, namely the dual-tree complex wavelet transform [20], [21] (see methods). In the following section we use simulated and real world images to compare the performance of this new method with more classical ones. We show that, thanks to our method, a fairly unbiased test of pairwise association is now achievable despite the presence of strong and long-range autocorrelation within one or both of the variables involved. We address not only the case of continuous data but also of binary ones.

## Results

**Figure 1:**
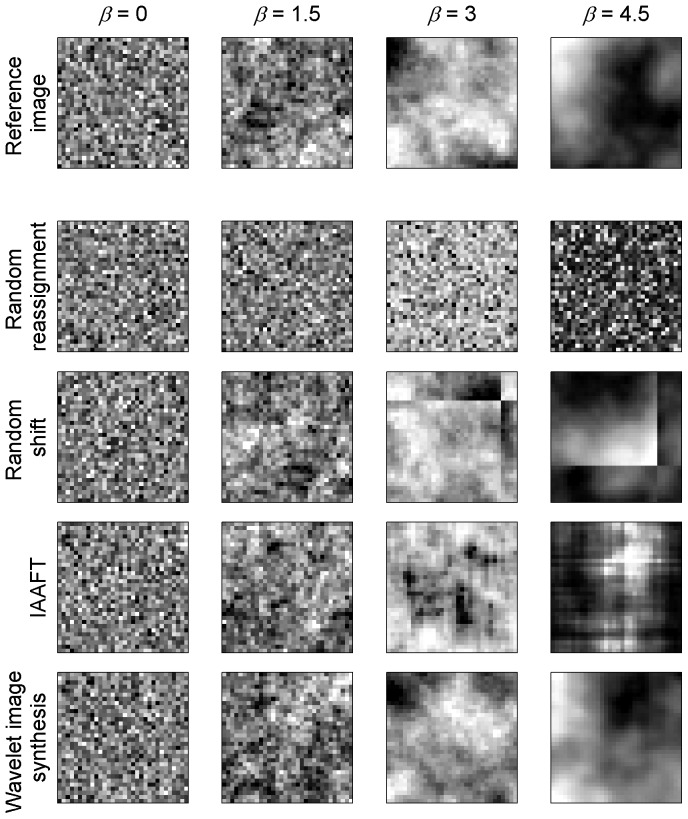
Examples of surrogates for images with increasing degree of autocorrelation. The first row features particular simulations of fractal patterns (fractional Brownian field) generated by Fourier synthesis [27] for four degrees of autocorrelation (from short- to long-ranged as indirectly quantified by the *β* parameter). The following rows (2 to 5) display one particular random realization (i.e. re-simulation) of each of these fractal patterns according to four surrogate producing methods (random reassignments, random shifts, iterative amplitude adjusted Fourier transform (IAAFT) and wavelet-based energy synthesis, respectively).

The Monte-Carlo test consists in assessing the significance of an observed test statistic by comparing it with a set of values of this statistic obtained by generating random data, called “surrogates” in signal processing, using some assumed model [22], [23]. Because the surrogate generation process attempts to implement a null hypothesis, we then refer to it as a “null model” [24]. In Figure 1, examples are given for random realization of the four null models considered here with respect to four simulated fractal images with increasing degrees of autocorrelation (as controlled by the energy spectrum exponent *β*, see methods).

The first null model is a complete random reassignment of pixel values within the image (Fig. 1, row 2). This randomization not only destroys the link between the paired observations of the two variables (which is desirable to test association) but also destroys the possible autocorrelation pattern in each variable. This non-spatial null model has obvious drawbacks and is just included here for comparison.

The second model is the random shift null model which consist of random rotation, reflection, and translation in the X- and Y- Cartesian axes [25], [26] (Fig. 1, row 3). Values that are shifted beyond the edge of the data grid are wrapped back on to the opposite edge as if the data was actually mapped onto a torus. While preserving the general autocorrelation structure of the pattern, this procedure however introduces artificial linear frontiers by placing at adjacent locations values initially located at opposite edges. In the case of highly autocorrelated data, the shifting procedure therefore artificially introduces low autocorrelation values at short distances between the adjacent sides of the shifted edges.

The third null model is known as the iterative amplitude adjusted Fourier transform (IAAFT) (Fig. 1, row 4). The IAAFT surrogates are widely used in hypothesis testing because they can be efficiently generated and they preserve both the frequency distributions and the power spectra (and hence the autocorrelation) of the original data while at the same time being realizations of random processes [23]. A detailed description of this method is provided in the methods section. However, the Fourier transform implicitly considers the signal as an infinite periodic sequence, and, therefore, may underestimate the autocorrelation function at small scales for similar reasons as the random shift procedure does.

We propose here a fourth null model inspired by the IAAFT, yet benefitting from recent developments in image synthesis techniques. It is based on the dual-tree complex wavelet transform (DT-CWT) [20], [21], which is an improvement on classical discrete wavelet transform techniques (DWT) that is immune to shift dependence, i.e., major variations in the distribution of DWT coefficient energy over scales and orientations caused by small integer displacements of the pattern. DT-CWT also has the advantage of a better distinction and reproduction of possible pattern directions. Lastly, like most wavelet approaches, it is immune to the aforementioned IAAFT bias (Fig. 1, row 5). See the methods section for a full description of the procedure.

In order to determine whether the above null models are able to provide unbiased association tests between two autocorrelated variables, we assessed the control of the type I error as described in [8], [15]. The first step is to generate one pair of independent reference images to be tested for dependence. Both images are generated with the same degree of autocorrelation using Fourier synthesis (see methods and reference images in Fig. 1). This Fourier synthesis generates self-similar, or fractal, spatial structures, which is a desirable property because these are common in nature and especially in natural landscapes, as they often arise when a single structuring process dominates over many scales [27]–[29]. For each of the above null models, 499 pairs of Monte Carlo replicates were then generated from the actual pair (see [22] for a discussion about the adequate number of replications). Pearson's product-moment correlation coefficient (referred to as the *r* statistic) was then computed for each of the 500 pairs, including the actual one, so as to build the null probability distribution function (PDF) of the test statistic. For a one sided-test, large values of the observed test statistic provide evidence against the null hypothesis. The statistic is declared significant at the level α if it is one of the largest *n* α values (e.g. 25 if α = 5% and n = 500). In the case of Pearson's product-moment correlation coefficient, which may vary between −1 to 1, the test is two-tailed and we consider the proportion of simulations for which the correlation *r_sim_* falls outside the interval ]- *r_obs_*, *r_obs_*[ where *r_obs_* is the observed correlation. This statistical test was repeated on 1000 pairs of reference images. The proportion of reference pairs declared to be significantly correlated (observed type I error rate) was assessed over the full possible range of significance levels α [0,1] (expected type I error) as to compute a type I error calibration curve allowing the comparison between null models and/or reference data. Note that we do not deal here with the power of statistical tests (the probability of rejecting the null hypothesis when the null hypothesis is not true) which is related to the type II error. Along with the aforementioned null models, we also computed Dutilleul's modified *t*-test [18], [19], which assesses the “effective” number of degrees of freedom in autocorrelated data. This method assumes the existence of a maximal range for the autocorrelation in order to analytically derive the variance of the sample covariance between the two variables.

**Figure 2:**
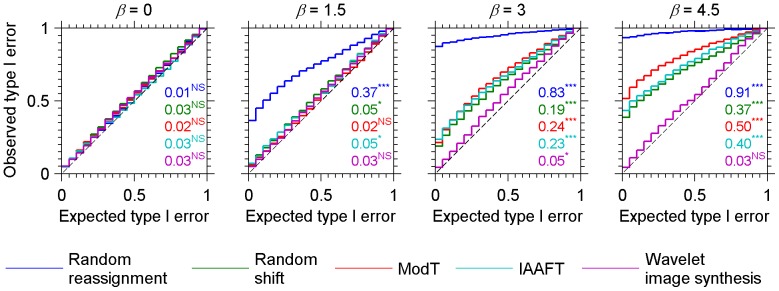
Type I error calibration curves for continuous fractal patterns generated through Fourier synthesis of 32×32 pixels images [27] (see Fig. 1 for examples). Observed versus expected type I error probabilities resulting from independence tests on 1000 pairs of simulated images, are plotted for every combination of method (colour curves) and degree of spatial autocorrelation (panels) as measured by the energy spectrum exponent, *β*. Methods compared are random reassignments, random shifts, corrected *t*-test of Dutilleul (ModT), iterative amplitude adjusted Fourier transform (IAAFT), and wavelet-based image synthesis. Each bin is 0.05 wide. The Kolmogorov-Smirnov (K.-S.) maximum difference statistic which measures the departure from the line of identity (dashed line) is indicated for each curve along with results of the derived test of the departure: *** = *p*-value<0.001; * = 0.01≤*p*-value<0.05; NS = not significant.

Type I error calibration curves for four different degrees of spatial autocorrelation and for the aforementioned null models are presented in Figure 2. When spatial autocorrelation is absent (*β* = 0, i.e. white noise) from both maps involved in the spatial dependence test, all the four null models along with the corrected test of Dutilleul generated type I error rates that were non-significantly different from expected (Fig. 2.). This indicates that when there is no spatial dependence within the data, any of the above null models can be the basis of a valid statistical test.

With increasing spatial autocorrelation (*β*>0, i.e. colored noise), the random reassignment null model shows an increasing inflation of type I error rates at the classical critical significance level of 0.05 which is used in life and social sciences. This inflation means that a significant association will be falsely concluded more often than the nominal 5%. The inflation is confirmed by the high values and strong significance of the associated Kolmogorov-Sminorv maximum difference statistic (d_MAX_). The inflation is very high even at a fairly low degree of spatial autocorrelation (*β* = 1.5) for which we found that an erroneous significant association would be concluded over 36% of the time instead of the expected 5%. This result is consistent with previous findings that the traditional statistical tests, conceptually based on random reassignment, generates unacceptably high rates of type I error in presence of SA [12], [13].

For the three other null models, good control of type I error were obtained for the lower degrees of SA considered. However, the inflation in type I error rates started to be substantial for *β*≥3. For the random shift and IAAFT null models, the inflation was related to the loss of spatial autocorrelation between values on both sides of the shifted edges. This loss results in the inability of the null model to correctly account for the presence of high degrees of spatial autocorrelation extending over distances far larger than the image size, and therefore induces a marked type I error rate inflation.

The Dutilleul's modified *t*-test showed even stronger inflation of type I error for *β*≥3. It is indeed not suited for fractal or scale-invariant patterns, that feature spatial dependence at every spatial scale (also known as long-memory processes), and therefore violates one of the central assumptions of this test, that postulates vanishing SA beyond a certain range.

The wavelet-based synthesis null model gave very good type I error control for every considered degree of autocorrelation. Varying image sizes from 4 to 128 pixels (results not shown here) did not affect the type I error calibration curves, thus suggesting that the validity of this method is not affected by image size.

**Figure 3:**
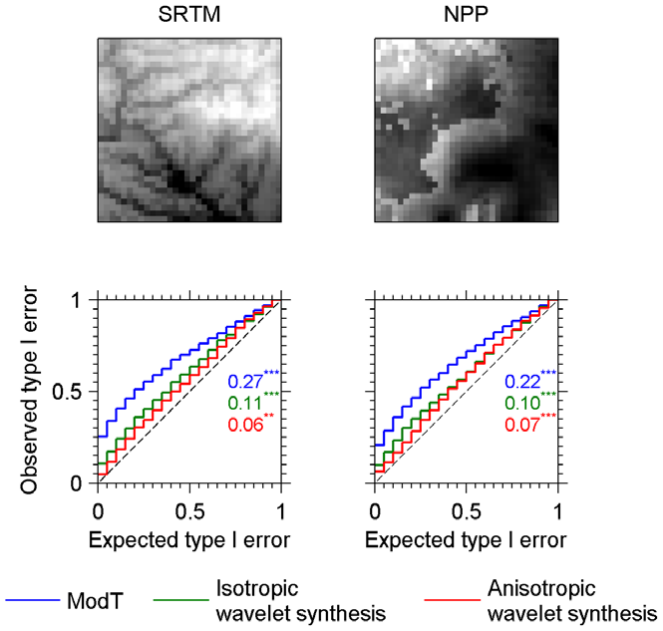
Type I error calibration curves for real data pertaining to earth relief or biomass production. Observed versus expected type I error probabilities resulting from an independence tests on 1000 pairs of simulated images, are plotted for every combination of method (colour curves) and dataset (panels). Methods compared are the corrected *t*-test of Dutilleul (ModT) and wavelet-based image synthesis method, with direction selectivity feature disabled (isotropic) and enabled (anisotropic). Data are non-overlapping windows extracted either from digital elevation models (SRTM 1.3°×1.3° windows) or net primary production map (NPP 2.3°×2.3° windows). The first row of the figure features particular extracts exemplifying the kind of patterns characteristic of each dataset. Each bin is 0.05 wide. The Kolmogorov-Smirnov (K.-S.) maximum difference statistic which measures the departure from the line of identity (dashed line) is indicated for each curve along with results of the derived test of the departure: *** = *p*-value<0.001; ** = 0.001≤*p*-value<0.01.

Natural systems are generally not truly fractals because multiple structuring processes that induce different spatial patterns at different scales may occur. We therefore also tested the validity of the wavelet-based image synthesis null model against actual patterns. In order to get a large number of independent real-world lattice data to assess the relevance of the null models, we subdivided a world-wide lattice dataset of altitude above mean sea level into non-overlapping grids of 32×32 cells. This dataset comes from the Shuttle Radar Topography Mission (SRTM), which obtains elevation data using a radar system flown onboard a space shuttle [30]. Each value in the lattice is averaged over a square area of 2.5 arc-minutes. We applied the same procedure on a global map of net primary production (NPP, carbon m^−2^ year^−1^) which is derived from the Advanced Very High Resolution Radiometer satellite images [31]. In our case, the meaning of the values is less important than the spatial structure occurring within the datasets. The altitude dataset is characterized by rather smooth variations in space and near self-similarity. The productivity dataset differs from the SRTM by frequent abrupt transitions often linked to changes in land covers. Figure 3 indicates that the wavelet-based synthesis null model performs well not only on stochastic fractal maps but also on real-world patterns with either smooth (SRTM) or abrupt variations (NPP). In order to evaluate the efficiency of the direction selectivity property (anisotropy) of the wavelet-based image synthesis, we implemented an alternative version of the null model where direction selectivity is disabled. This modification is achieved by computing and matching the energies at the scale level rather than separately for each of the six direction subbands (see methods). The resulting direction independence (isotropy) leads to a doubling of type I error rates at α = 0.05 (Fig. 3), which shows that direction selectivity is a decisive property of the surrogate generation process to achieve an unbiased test.

**Figure 4:**
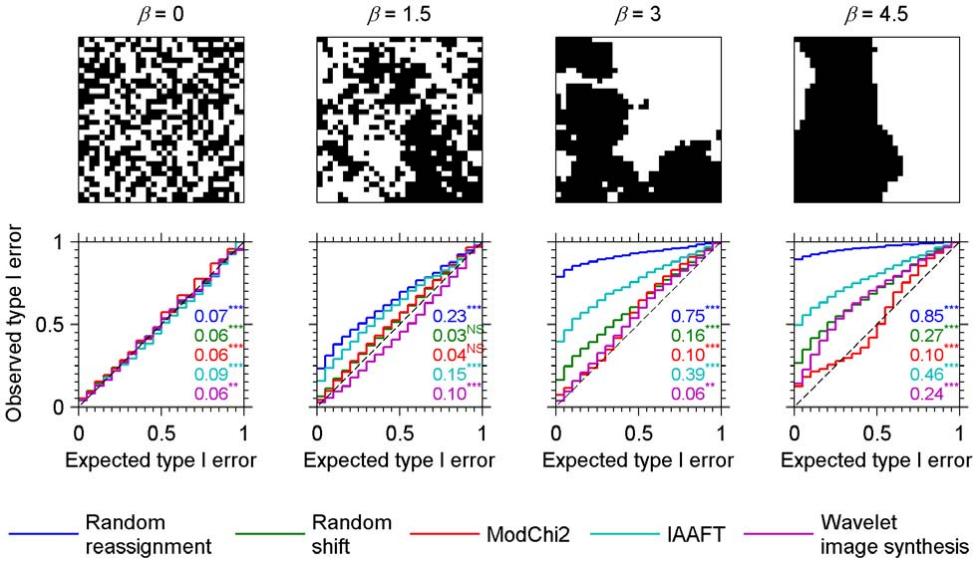
Type I error calibration curves for fractal binary data generated through Fourier synthesis and thresholding of 32×32 pixels images [**27**] **.** Observed versus expected type I error probabilities resulting from an independence tests on 1000 pairs of simulated images, are plotted for every combination of method (colour curves) and degree of spatial autocorrelation (panels) as measured by the energy spectrum exponent, *β*. Methods compared are random reassignments, random shifts, modified chi-square test of Cerioli (ModChi2), iterative amplitude adjusted Fourier transform (IAAFT), and wavelet-based image synthesis. Each bin is 0.05 wide. The Kolmogorov-Smirnov (K.-S.) maximum difference statistic which measures the departure from the line of identity (dashed line) is indicated for each curve along with results of the derived test of the departure: *** = *p*-value<0.001; * = 0.01≤*p*-value<0.05; NS = not significant.

We finally tested the methods against binary images (Fig. 4) generated by thresholding the simulated continuous-valued fractal patterns (see methods). In this case, a classical statistic of association between two binary patterns is Pearson's chi-square, which ranges between [0+∞] so that a one-tailed test of association is required. We also used the modified Pearson's chi-square statistic proposed by Cerioli [32], [33] for autocorrelated discrete-valued spatial processes in lieu of Dutilleul's. Here also, increasing inflation of type I error with increasing spatial autocorrelation were found for the random reassignment and IAAFT null models as reported by [8] for the former. The random shift null model was able to handle a degree of autocorrelation as high as *β* = 1.5, but failed in face of higher degrees of spatial autocorrelation. The modified test of Cerioli and the wavelet-based null model showed acceptable results for *β* up to 3. But; although the inflation was about two times lower than observed for the random shift null model, both formed a quite liberal test for strong SA (type I error of about 0.13 instead of 5% with *β* = 4.5). For the wavelet-based null model, these inaccuracies are the consequence of the supplementary constraint of matching frequency distribution which inevitably weakens the matching of energies (SA function).

## Discussion

Null models are tools for determining how strongly associated two variables would be if associations were only caused by coincidental similarity. If the observations are not the result of independent experimental treatments, but rather sequential observations in space, the null model should incorporate the autocorrelation likely to be present within the variables because it is a major source of coincidental covariations.

Our result underscores the need to verify prior to any analysis that the data are in agreement with the particular assumptions of each null model. The random reassignments null model, which assumes complete spatial randomness, is generally inappropriate and yields biased results as already noted by many authors. For the random shifts null model, complete spatial randomness is not assumed, but there is an assumption of periodical boundaries, which is highly unrealistic for geographical observations, and becomes a source of type I error rate inflation if autocorrelation is long-ranged. The modified *t*-test of Dutilleul and the modified chi-square test of Cerioli both make the assumption of long-range independence of observations. As signalled by the increased type I error rate we observed on real images, this assumption is limiting for the use of these corrected tests regarding a wide class of real-world data of ‘smooth’ aspect.

For this reason, we propose a wavelet-based null model aiming to synthesize image data while conserving the observed autocorrelation function in the form of the wavelet energy spectrum. Here we show that this null model, that makes no assumptions regarding the shape of the autocorrelation function, is far less sensitive to the presence of long range dependence than other methods. Since no serious bias was noted with both synthetic and actual data we conclude that the method is a sound basis for association tests involving any kind of continuous-valued lattice data (maps, images).

As shown by the present results the wavelet image synthesis null model is also relevant to gridded binary data. Indeed, bias in the type I error distribution remains absent or acceptable as long as the pattern does not tend too much towards the “red-spectrum” type, i.e., fractal patterns with very high degree of autocorrelation, say *β* values above four. For values under that threshold, it appears that the method can be safely applied to test the association between two binary images or between a binary and a continuous variable (result not shown).

The method is however limited to lattice data and cannot be applied to data resulting from a sampling design, for which case we have verified here that Dutilleul's or Cerioli's methods remain good options as long as the risk of bias due to long range correlations is borne in mind.

We presented here an application of our wavelet-based image synthesis null model for the study of multi-scale pairwise association. The functioning of the null model is however not limited to the use of Pearson's *r* and analogous chi-square statistics but can be ported to any test statistic derived from two or more lattice data. For instance, inferences on scale-specific correlation or “causality” [17]–[19] imply the use of surrogate data. Association tests are also promising tools for the validation of species distribution models (SDMs, also known as ecological niche modeling), which combine observed species' occurrences with mapped environmental data to predict species probability of occurrence at unsampled locations [3]. A common practice for evaluating SDMs predictive ability involves the computation of accuracy statistics on an independent set of occurrences [34]. However, the production of truly independent additional observations is highly challenging due to the autocorrelated nature of both species' distributions and environmental drivers, thus leading frequently to over-optimistic estimates of models' predictive ability [35]–[37]. Moreover, even if an independent sample is available, correspondence between predictions of the model and additional occurrences may arise by chance for the very reasons already mentioned for association tests and, thus, this does not prove the validity of the model but merely our failure to disprove it. The rejection of the null hypothesis, that the species presence is indifferent to the environmental factors considered, requires the construction of a null model. By generating the probability distribution of any accuracy statistic expected for random association between environmental drivers and species presence, one can then decide whether or not to reject the null hypothesis. For this aim, several statistical approaches have been proposed [38]–[40] but none of them consider the spatial location of samples and the probable autocorrelation. More recently, Raes and ter Steege [7] addressed the autocorrelation within a species' occurrence map created by a heterogeneous sampling effort. To account for it, they proposed a null model approach based on the random reassignment of the occurrence map. SA was incorporated by restricting the randomly drawn occurrences to localities known to be part of the sampling scheme of the study area (i.e. the entire set of sampled occurrences irrespective of the species label). Regarding this issue, the wavelet-based image synthesis null model for the simulation of binary occurrence maps has the advantage of reproducing the autocorrelation patterns either linked to sampling or to species' dynamics. At no other expense than an increase in processing time, this null model could be incorporated into a significance test for any test statistic (e.g. the widely used area under the curve or AOC [34]) without the need to gather data on sampling effort across the study area that are most often unavailable.

Considering the realm of applications that rely on lattice data, there are two main directions in which to develop the approach of wavelet-synthesis null model. First, it seems quite straightforward to extend the method to assessing the relative influence of explanatory variables on a response variable through coefficients of partial correlations. Second, there is a need to consider lattice data which are partially censured, for example maps or regions or borders of a continent along a shoreline. Particular algorithms for the estimation of the power spectrum from irregularly sampled data might provide an answer but this remains an open field of investigation.

## Methods

### 1. Replicating image autocorrelation function using Fourier transform

The properties of the discrete Fourier transform (DFT), have widely been used for the generation of surrogates which preserve the “linear properties” of the data (the first and the second moments, i.e. mean, variance and auto-correlation functions) [23], [41], [42]. Fourier analysis consists in representing any function in terms of the sum of its projections onto a set of sinusoid functions of discrete frequencies and directions. The decomposed function in frequencies i and j, in X and Y Cartesian directions, can be expressed as *a_ij_* exp(*i Φ_ij_*) with a magnitude *a_ij_* and a phase *Φ_ij_* (the displacement of each sine wave from an arbitrary common origin). From the magnitude and phase coefficients the original signal can be exactly reconstructed by the inverse discrete Fourier transform (IDFT). The squared values of the magnitude coefficients make the power spectrum or energy spectral density of the original signal, and corresponds to the decomposition of the variance (or “energy”) of the signal into harmonic frequencies. In order to generate random maps that all match the autocorrelation structure of a given map, a straightforward way is to take advantage of the Wiener–Khinchin theorem [43] which states that the energy spectral density of a stationary random process is the Fourier transform of the corresponding autocorrelation function. This means that any set of maps sharing the same energy spectral density also shares an identical autocorrelation function and vice-versa. A simple phase randomized surrogate will therefore achieve our goal of preserving the autocorrelation function. However, the data generated this way will have a normal distribution. Because the normality assumption is rarely met by actual data, a method was proposed in [44] that preserve both the sample probability distribution of values and the power spectrum (hence autocorrelation function). This method is known as iterative amplitude adjusted Fourier transform (IAAFT):

1. Randomly shuffle the values in the empirical data.
2. Convert the data into the frequency domain by computing its two-dimensional discrete Fourier transform.
3. Conserve the phases *Φ_ij_* of the Fourier coefficients and replace the magnitudes *a_ij_* with those of the original data.
4. Form the complex coefficients that encodes both the magnitude and the phase component *a_ij_* exp(*i Φ_ij_*) ≡ *a_ij_* cos *Φ_ij_*+i *a_ij_* sin *Φ_ij_*;
5. Convert back to the spatial domain by taking the inverse Fourier transform of the coefficients and drop the imaginary part to convert the complex-valued result to real values.
6. Because the probability distribution will no longer be correct, transform the data to the initial probability distribution by rank ordering and replacing each value with the value in the original data having the same rank.
7. Repeat steps 2 to 6 until the power spectrum adjustment step no longer alters the rank order.

The procedure could be terminated with either step 5 or 6, depending on whether the residual biases are more tolerable in the power spectrum or in the probability distribution, respectively [44]. Here we choose the probability distribution matching as the final step. However, choosing the alternative has no substantial influence on our results (not shown). Besides its wide availability in software packages, the Fourier transform has several important properties including rapid computation time, shift invariance (a translation of the signal does not affect the energy of the Fourier coefficients), and directional selectivity. However, the Fourier analysis implicitly treats the signal as an infinite periodic sequence (known as periodic extension), as if the map were wrapped around a torus. The eventual abrupt transitions that occur between opposite edges are translated in the frequency domain into undesirable aliasing frequencies. This undesirable noise in the spectral estimates can be reduced by either windowing or detrending the data prior to decomposition. However, the low frequency content of the windowing or trend functions will introduce an additional bias. As a consequence, to be valid as a null model, the Fourier transform procedure requires assuming an infinite periodic signal, as does the random shift null mode, which is unrealistic in many situations.

### 2. Replicating image autocorrelation function using wavelet transform

Also appropriate for the estimation of the power spectrum, are the locally oscillating basis functions known as wavelets. They have widely been used for the generation of surrogate data in one dimension [45]–[47]. An intuitive way to understand the wavelet transform is to imagine that we shift a given wave template contained in a window so as to center it on top of every value along the X- and Y-axis of the image. The transform produces a grid of coefficients, known as a subband in the wavelet jargon, with higher amplitude when the portion of the image in the gliding window matches the wave template in form and dimension, and lower amplitude when it does not. The image is then down-sampled by a factor of two and this convolution with the wave template is performed again to get the subband corresponding to the next coarser scale. The wavelet transform thus provides a space-frequency analysis of the signal by measuring its frequency content at every point in space. When processing coefficients near the edges of the image data, the transform will require samples outside the defined range. Unlike with the DFT which demands a periodic infinite extension of the signal (as if the image is wrapped on a torus), the wavelet transforms can be implemented with so called symmetric extension, which adds values following a mirror symmetry around the edges of the original data. In this way, the signal is extended without any artificial abrupt transition. Unfortunately, in spite of its efficient computational algorithm, the traditional discrete wavelet transform (DWT) suffers from three interrelated shortcomings that are not present in the Fourier analysis [48]. First, the transform exhibits shift dependence, i.e., small integer displacements of the pattern can cause major variations in distribution of DWT coefficient energy over scales and orientations. Second, the inverse transform achieves imperfect reconstruction of the original image and the shift dependence leads to artifacts (aliasing) if some coefficient processing is applied. Finally, it has poor directional selectivity in two or more dimensions, i.e., it cannot distinguish frequencies on opposing diagonals (±45°).

A common way to overcome these shortcomings is to use Gabor filters. However, the transformation to the Gabor space is computationally expensive and the high redundancy in the output coefficients increases the complexity of subsequent processing. Fortunately, the Q-shift dual-tree complex wavelet transform (DT-CWT), a recent enhancement to the DWT [20], [21], overcomes these shortcomings. Thanks to a moderate increase in redundancy (2×D redundancy for D-dimensional signals) and computational load, this complex wavelet transform inspired by the Fourier representation comes very close to mirroring the attractive properties of the Fourier analysis [48]: a nearly shift-invariant magnitude; a substantially reduced aliasing; and directional selectivity in two or more dimensions. In practice, a 2D CWT produces six complex-valued subbands at each scale, which are oriented at angles of ±15°, ±45°, ±75°. For a good tutorial overview on the DT-CWT we refer the reader to Selesnick et al. [48].

We propose the use of the following procedure, first given by de Rivaz ([49] p. 60–65) as an image synthesis technique, in order to generate images sharing a given energy spectrum, and hence a 2D autocorrelation function:

1. Center and normalize the reference image *A*.
2. Generate a normal white noise *B* of mean 0 and variance 1 of the same size as *A*;
3. Use the DT-CWT with symmetric extension (we choose half-point) to generate the multi-scale and multi-orientation decompositions of both *A* and *B*. We chose the (13,19)-tap near-orthogonal filters at scale 1 and the 14-tap Q-Shift filters at scales ≥2 because it is a good compromise between computational complexity and aliasing energy [50].
4. Scale the magnitude of the detail coefficients of each subband of *B* so that the subband energy (the summed squared magnitude of all subband coefficients) is equal to the corresponding energy of the *A* subbands. If the original energy is *E_A_* and the desired energy is *E_B_* then the correct scaling factor is 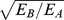. Leave the coarsest scale coefficients (approximation subband) unchanged.
5. Transform the scaled coefficients of B back into the spatial domain.
6. Repeat steps 3 to 5 a sufficient number of times for good correspondence of the subbands energy (we find 25 iterations to be sufficient for a 32×32 pixels map).

Iteration is needed because the value taken by the DT-CWT coefficients are not independent through scales and directions, i.e. there is some redundancy in the information. The DT-CWT is performed up to the maximum possible level of decomposition (log_2_ of image size) in order to reproduce the autocorrelation occurring at every scale. In this way, we construct surrogates images and a null model devoid of any assumption regarding the shape of the autocorrelation function and the existence of particular orientations in the pattern (anisotropy). Examples of wavelet-based image synthesis from continuous fractal patterns (see below) of varying degrees of autocorrelation are presented in Fig. 1.

Ecological variables often consist of discrete data such as in the analysis of species co-occurrence patterns. The wavelet-based image synthesis is able to generate discrete data by adding to the energy matching procedure the supplementary constraint of conserving the frequency distribution of gray levels of the reference image *A* which, in the case of discrete binary images, simply reflects the relative abundances of one and zero values. With this aim, we match the frequency distribution of *B* to the frequency distribution of the centered and normalized reference image *A* in the spatial domain (before step three and after step five in the above algorithm). The matching of the frequency distribution is computed by means of two tables. The first table is computed at each iteration and gives the correspondence between the values in *B* and their rank order. The second table is computed once for all iterations, and gives the correspondence between rank order and value of the reference image. In order to avoid local minima in the convergence process, we add some random white noise in the wavelet domain before step 4.

### 3. Generating reference images with fractal pattern through Fourier synthesis

A subclass of two dimensional self-similar random processes which is easy to generate is the fractional Brownian field (fBf) that was introduced by Mandelbrot and Van Ness [51].

True fBf is a normally distributed, zero mean, non-stationary isotropic stochastic process with stationary increments and energy spectral density, *S*, that is a power function of the frequency, *f*:
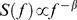
(1)


The energy spectrum exponent *β* is directly related to the fractal dimension of the map [27] and controls its degree of autocorrelation. A large *β* introduces relatively smooth, correlated, variations into the map, known as colored noise, whereas *β* = 0 (flat spectrum) results in a rough uncorrelated sequence known as white noise. With *β*>0, as the energy of spectral components increases monotonically with frequency, there is no range beyond which the autocorrelation vanishes.

An infinite number of fBf maps may be generated for a given *β* by constructing a frequency space complying with eq. 1 and randomly choosing the phase of each sine wave before applying the IDFT [27], [52]. It is related to the IAAFT described above but directly based on eq.1 instead of the spectrum of a reference image. Yet this efficient procedure actually generates an approximated fBf because it yields stationary processes that have periodic boundaries (i.e. the fBf is mapped onto a torus) and are therefore not fully self-similar [53]. We circumvented the undesirable high correlations between observations at opposite edges despite the long distance by producing fBf lattices of 128×128 values, and only retaining a 32×32 section. Examples generated in this way are presented in Fig. 1 for different *β*. Note that the computation of true fBf could have been achieved via a Cholesky-Levinson factorization [54]. However, this method requires an immense computational and memory load [53], and exact self-similarity is not necessary for this work since our purpose is simply to obtain simulated maps with a pre-defined autocorrelation function.

In order to generate binary maps, we merely converted the above-described continuous fBf into 0 and 1 by taking the median as a cutoff value. Examples of binary maps with increasing degree of SA are presented in Fig. 4.

The implementation of the method for the generation of surrogates is available online as Java archive S1.

## Supporting Information

Java archive S1 **Implementation in Java 1.7 of the wavelet-based method described in this paper for the generation of two dimensional surrogates.**
(JAR)
